# Pre-percutaneous coronary intervention sudden cardiac arrest in ST-elevation myocardial infarction: Incidence, predictors, and related outcomes

**DOI:** 10.3389/fcvm.2023.1100187

**Published:** 2023-02-16

**Authors:** Guilherme Pinheiro Machado, Andre Luiz Theobald, Gustavo Neves de Araujo, Anderson Donelli da Silveira, Rodrigo Vugman Wainstein, Julia Fagundes Fracasso, Matheus Niches, Angelo Chies, Sandro Cadaval Goncalves, Mauricio Pimentel, Marco Vugman Wainstein

**Affiliations:** ^1^Postgraduate Program in Health Sciences: Cardiology and Cardiovascular Sciences, Universidade Federal do Rio Grande do Sul, Porto Alegre, Brazil; ^2^Department of Cardiology, Hospital de Clínicas de Porto Alegre, Porto Alegre, Brazil; ^3^Imperial Hospital de Caridade, Florianópolis, Brazil; ^4^Instituto de Cardiologia de Santa Catarina, São Jose, Brazil; ^5^School of Medicine, Universidade Federal do Rio Grande do Sul, Porto Alegre, Brazil

**Keywords:** ST-elevation acute myocardial infarction, sudden cardiac arrest, percutaneous coronary intervention, cardiogenic shock, mortality

## Abstract

**Background:**

ST-segment elevation myocardial infarction (STEMI) is a frequent cause of sudden cardiac arrest (SCA) and early percutaneous coronary intervention (PCI) is associated with increased survival. Despite constant improvements in SCA management, survival remains poor. We aimed to assess pre-PCI SCA incidence and related outcomes in patients admitted with STEMI.

**Methods:**

This was a prospective cohort study of patients admitted with STEMI in a tertiary university hospital over 11  years. All patients were submitted to emergency coronary angiography. Baseline characteristics, details of the procedure, reperfusion strategies, and adverse outcomes were assessed. The primary outcome was in-hospital mortality. The secondary outcome was 1-year mortality after hospital discharge. Predictors of pre-PCI SCA was also assessed.

**Results:**

During the study period 1,493 patients were included; the mean age was 61.1 years (±12), and 65.3% were male. Pre-PCI SCA was present in 133 (8.9%) patients. In-hospital mortality was higher in the pre-PCI SCA group (36.8% vs. 8.8%, *p* < 0.0001). In multivariate analysis, anterior MI, cardiogenic shock, age, pre-PCI SCA and lower ejection fraction remained significantly associated with in-hospital mortality. When we analyzed the interaction between pre-PCI SCA and cardiogenic shock upon admission there is a further increase in mortality risk when both conditions are present. For predictors of pre-PCI SCA, only younger age and cardiogenic shock remained significantly associated after multivariate analysis. Overall 1-year mortality rates were similar between pre-PCI SCA survivors and non-pre-PCI SCA group.

**Conclusion:**

In a cohort of consecutive patients admitted with STEMI, pre-PCI SCA was associated with higher in-hospital mortality, and its association with cardiogenic shock further increases mortality risk. However, long-term mortality among pre-PCI SCA survivors was similar to non-SCA patients. Understanding characteristics associated with pre-PCI SCA may help to prevent and improve the management of STEMI patients.

## Introduction

Sudden cardiac arrest (SCA) is a devastating manifestation of ischemic heart disease. Most patients do not survive hospitalization and mortality rates range between 40 and 60 (1, 2). Primary percutaneous coronary intervention (PCI) improves outcomes and is the first-line strategy in patients with ST-segment elevation myocardial infarction (STEMI) (3, 4). Despite advances in pre-hospital care and improvement of PCI techniques, mortality in STEMI-related SCA remains high.

Although the cause of SCA is ischemic heart disease in up to 70% of patients, the etiology is often unclear immediately after the index event, in particular, considering that an electrocardiogram (ECG) may not be reliable after the return of spontaneous circulation (5, 6). A recent randomized controlled trial showed no benefit of early coronary angiography in patients with out-of-hospital cardiac arrest and no ST-segment elevation on ECG (7). However, in the presence of ST elevation on post-SCA ECG, early invasive stratification is mandatory according to STEMI guidelines (4).

Understanding characteristics associated with SCA may help to prevent and improve the management of STEMI patients. We aimed to assess pre-PCI STEMI-related SCA incidence, predictors, and related outcomes in patients admitted to a tertiary public hospital in southern Brazil.

## Methods

### Study design and patients

This was a prospective single-center cohort of consecutive patients with STEMI admitted for primary PCI at Hospital de Clínicas de Porto Alegre, a tertiary public teaching University Hospital in southern Brazil, between March 2011 and September 2022. Patients eligible for inclusion were consecutive adults (≥18 years of age) with suspected STEMI, based on the presence of typical chest pain at rest associated with ST-segment elevation or abnormalities that met the diagnostic criteria for STEMI. STEMI diagnosis and treatment were defined according to the latest guidelines available at the time of study enrolment (4, 8). Exclusion criteria were non-ST elevation myocardial infarction (NSTEMI), another final diagnosis, and myocardial infarction with non-obstructive coronary arteries. All patients provided written informed consent this prospective cohort was approved by the Research Ethics Committee. Medications and PCI strategies were based on current guidelines and performed according to the operator’s choice. Manuscript writing was guided by the STROBE checklist (9).

### Clinical data and outcomes

Clinical data were collected during the hospital stay and included: baseline clinical characteristics, medical history, and procedure characteristics. The primary outcome was predictors of in-hospital mortality. The secondary outcome was 1-year mortality after hospital discharge. Long-term follow-up was ascertained by clinical visits or telephone contact with patients or their families. Time-to-event was expressed in months.

### Definitions

Pre-PCI sudden cardiac arrest was defined as cardiac arrest occurring before catheterization and primary PCI (before the first contrast injection in a coronary artery) and requiring resuscitation procedures (i.e., ventilation, chest compression, and defibrillation). Patients who were not submitted to primary PCI after cardiac catheterization (e.g., small vessel with distal occlusion, unsuccessful procedure, or indication for CAB) were also included.

Cardiogenic Shock (CS) was defined as systolic blood pressure ≤ 90 mmHg for more than 30 min, or need for inotrope/vasopressor support, signs of end-organ failure (clammy skin, capillary filling time > 3 s, urine output <0.5 mL/kg/h, lactate level > 2 mmol/L), or low cardiac output (<2.2 L/min/m^2^ if treated with inotropes/vasopressors or < 1.8 L/min/m^2^ without inotropes/vasopressor therapy) (10).

Chronic kidney disease (CKD) is defined as kidney damage or glomerular filtration rate (GFR) <60 mL/min/1.73 m (2) for 3 months or more, irrespective of cause (11). A positive family history of CVD was defined as a self-reported diagnosis of CVD that occurred (age of onset <55 years for men and < 65 years for women) in first-degree relatives (in parents, siblings, or children) (12). Bleeding complications were classified according to GUSTO classification. The GUSTO bleeding definition uses four categories: severe or life-threatening, moderate, mild, and none. Severe or life-threatening if they were intracerebral or if they resulted in substantial hemodynamic compromise requiring treatment. Moderate bleeding was defined by the need for transfusion. Minor bleeding referred to other bleeding, not requiring transfusion or causing hemodynamic compromise (13).

Echocardiography assessment was performed within 48 h of hospital admission according to hospital routine. Diastolic dysfunction was assessed according to recommendations of the American Society of Echocardiography and the European Association of Cardiovascular Imaging (14). Diastolic dysfunction grading 1, 2, and 3 were described as mild, moderate, and severe, respectively. Right ventricle dysfunction was graded according to fractional area change (15).

Procedural outcomes were also described. Successful procedure was defined as final TIMI 2 or 3 flow and residual stenosis <30%. No reflow was defined as suboptimal myocardial reperfusion through a part of coronary circulation without angiographic evidence of mechanical vessel obstruction. Distal embolization was defined as a distal filling defect with an abrupt ‘cutoff’ in one of the peripheral coronary artery branches of the infarct-related vessel, distal to the site of angioplasty.

### Statistical analysis

Continuous variables were expressed as mean (SD) or median (interquartile range) based on the presence of symmetrical and asymmetrical distribution, respectively. The normality of the distribution of each variable was assessed by the Shapiro–Wilk test. Categorical variables were expressed as relative and absolute frequencies. Differences between groups were compared using Student’s *t*-test or Mann–Whitney test as appropriate. The chi-square test or Fisher’s exact test was used for categorical variables. The primary and secondary outcome analyzes were using logistic regression to estimate odds ratios. Multivariate models were performed for predictors of in-hospital mortality and predictors of pre-PCI SCA. For each model, statistically significant risk factors in univariate analysis were included (*p* < 0.1), as well as other relevant variables that could be incremental based on clinical experience. Multivariate model were performed for predictors of in-hospital mortality and predictors of pre-PCI SCA. For the primary outcome, a model including the interaction between cardiogenic shock and pre-PCI was also performed. All hypothesis tests had a two-sided significance level of 0.05. The Kaplan-Meier analyzes and comparison using the log-rank test were performed using MedCalc Statistical Software version 14.8.1 (MedCalc Software, Ostend, Belgium). All remaining statistical analyzes were conducted using SPSS Statistics for Windows, Version 26.0. (IBM Corp., Armonk, NY, United States).

## Results

**Figure 1:**
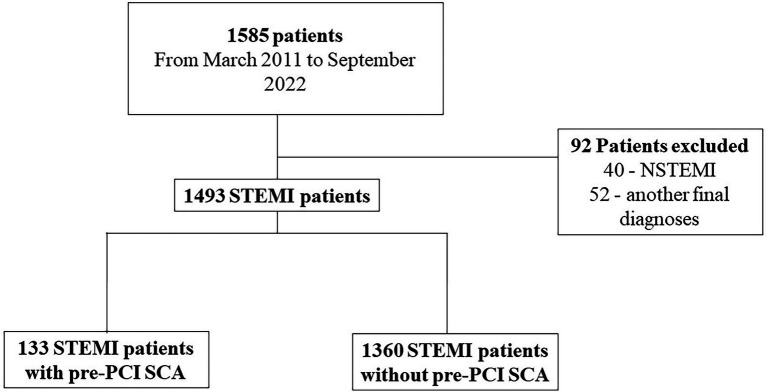
Flowchart.

Between March 2011 and September 2022, 1,585 consecutive patients admitted with suspected STEMI and referred for primary PCI were enrolled in our registry. Ninety-two subjects were excluded because had another final diagnosis, including NSTEMI. Pre-PCI SCA was present in 133 (8.9%) patients. Figure 1 shows the flow diagram of this study. The mean age was 61.1 ± 12 years; 65.3% of patients were male. Cardiogenic Shock at admission (52.6 vs. 5.8%; *p* < 0.0001), hypotension (26 vs. 5.8%; *p* < 0.0001), moderate/severe bleeding (7.5 vs. 1.9%; *p* < 0.0001) were more common in pre-PCI SCA patients. Post-MI ejection fraction and pain-to-door time were lower among Pre-PCI SCA patients (50 vs. 44%; *p* < 0.0001 and 210 vs. 300 min; *p* < 0.0001, respectively). Table 1 summarizes, respectively, the baseline characteristics of all patients. The number of patients for which the respective data was available is included in Supplementary material – Supplementary Table S1.

**Table 1:** Baseline characteristics of study patients.

Clinical characteristics	Without SCA (*n* = 1,360)	With SCA (*n* = 133)	*p-*Value
Age, years	61.3 (±11.8)	59.6 (±11.5)	0.11
Age ≥ 65 years	545 (40.1%)	44 (33.1%)	0.12
Male	885 (65.1%)	90 (67.7%)	0.54
BMI, kg/m^2^	27.03 (±4.8)	27.5 (±5.3)	0.28
Hypertension	852 (65.0%)	80 (60.2%)	0.26
Diabetes	372 (27.4%)	36 (27.1%)	0.94
Previous ASA use	234 (18.2%)	23 (17.4%)	0.96
Previous AMI	178 (13.1)	18 (13.5)	0.88
Previous PCI	152 (11.2%)	19 (14.6%)	0.24
Previous Stroke	111 (8.2%)	11 (8.3%)	0.98
Previous HF	33 (2.4%)	3 (2.3%)	0.90
Previous COPD	85 (6.3%)	8 (6.0%)	0.91
Previous CKD	63 (4.6%)	8 (6.0%)	0.17
Peripheral vascular disease	55 (4.0%)	3 (2.3%)	0.30
Family history	224 (16.7%)	20 (15.0%)	0.63
Smoking, current or previous	867 (63.7%)	72 (54.1%)	0.02
Atrial fibrillation	33 (2.4%)	6 (4.5%)	0.15
Temporary pacing	72 (5.3%)	21 (15.8%)	<0.0001
Cardiogenic shock	79 (5.8%)	70 (52.6%)	<0.0001
Admission status			<0.0001
Walking-in	279 (20.5%)	16 (12.0%)	
Ambulance emergency system	258 (19.0%)	47 (35.3%)	
In-hospital	35 (2.6%)	7 (5.3%)	
Transferred from other facility	787 (57.9%)	63 (47.4%)	
SCA location			
Out of hospital		71 (53.8%)	
In-hospital		50 (37.9%)	
Cath lab		11 (8.3%)	
Assisted SCA		125 (94%)	
Rhythm			
VF		79 (65.8%)	
Pulseless VT		10 (8.3%)	
Asystole		8 (6.7%)	
Pulseless EA		20 (16.7%)	
ECG after SCA			
STEMI		88 (84.6%)	
LBB		4 (3.8%)	
RBB		7 (6.7%)	
ROSC, min		14 (4–25)	
Conscious after resuscitation		21 (17.1%)	
Heart rate, bpm	80 [69–92]	90 [73–103]	<0.0001
SBP, mmHg	131 [114–154]	110 [87–129]	<0.0001
SBP < 90 mmHg	77 (5.8%)	34 (26.0%)	<0.0001
Pain-to-door, *min*	300 [180–480]	210 [104–381]	<0.0001
Door-to-balloon, *min*	72 [59–95]	80 [60–80]	0.153
Clinical characteristics	Without SCA (*n* = 1,360)	With SCA (*n* = 133)	*p-*Value
LVEF, *%*	50 [40–60]	44 [35–65]	<0.001
Diastolic disfunction			0.009
Mild	693 (56.7%)	50 (49.0%)	
Moderate	159 (13.0%)	9 (8.8%)	
Severe	18 (1.5%)	5 (4.9%)	
RV disfunction			0.01
Mild	34 (2.8%)	8 (7.8%)	
Moderate	8 (0.7%)	2 (1.9%)	
Severe	3 (0.2%)	1 (1.0%)	
Gusto bleeding classification			<0.0001
Minor	25 (1.8%)	5 (3.8)	
Moderate	8 (0.6%)	3 (2.3%)	
Severe	18 (1.3%)	7 (5.3%)	
Mechanical complication			0.90
Interventricular communication	2 (0.1%)	1 (0.8%)	
Left ventricle rupture	1 (0.1%)	0 (0.0%)	
Acute Mitral Regurgitation	1 (0.1%)	1 (0.8%)	
Dialysis in first 24 h of admission	10 (0.7%)	6 (4.5%)	0.0001
Thrombolysis			0.26
Rescue	11 (0.8%)	2 (1.16)	
Facilitated	32 (2.4%)	6 (4.7%)	
Pharmacoinvasive	8 (0.6%)	0 (0.0%)	
CABG	24 (1.8%)	2 (1.5%)	0.82
Arrythmias after 48 h			0.74
Atrial fibrillation	19 (1.4%)	2 (1.5%)	
Non sustained ventricular tachycardia	17 (1.3%)	3 (2.3%)	
Ventricular fibrillation	3 (0.2%)	0 (0.0%)	
Procedural characteristics			
Radial access	1,037 (76.5%)	56 (42.7%)	<0.0001
TIMI pre-PCI			0.98
0	857 (68.2%)	94 (80.3%)	
1	223 (17.8%)	11 (9.4%)	
2	98 (7.8%)	7 (6.0%)	
3	77 (6.1%)	5 (4.3%)	
TIMI post-PCI			0.002
0	40 (3.0%)	8 (6.3%)	
1	27 (2.0%)	3 (2.3%)	
2	87 (6.6%)	13 (10.2%)	
3	1,166 (88.3%)	103 (80.5%)	
Anterior wall MI	616 (45.3%)	76 (57.1%)	0.10
Infarct related artery			0.54
RCA	504 (37.3%)	43 (33.6%)	
LAD	603 (44.6%)	67 (52.3%)	
LCX	144 (10.6%)	10 (7.8%)	
LM	22 (1.6%)	4 (3.1%)	
Graft	8 (0.6%)	0 (0.0%)	
Procedural characteristics			
Number of vessels with severe stenosis			0.37
Single vessel disease	500 (36.8)	57 (42.9)	
Two vessel disease	417 (30.7)	36 (27.1)	
Three or more vessel disease	442 (32.5)	40 (30.1)	
No-reflow	85 (6.3%)	8 (6.0%)	0.91
Successful rates	1,255 (94.1%)	116 (91.3%)	0.20

SCA: sudden cardiac arrest; BMI: body mass index; ASA, acetylsalicylic acid; AMI, acute myocardial infarction; PCI, percutaneous coronary intervention; HF, heart failure; COPD, chronic obstructive pulmonary disease; CKD, chronic kidney disease; EA, electrical activity; VT, ventricular tachycardia; VF, ventricular fibrillation; STEMI, ST-elevation myocardial infarction; LBBB, left bundle branch block; RBBB, right bundle branch block; ROSC, return of spontaneous circulation; SBP, Systolic blood pressure; LAD, left anterior descending coronary artery; LCX, left circumflex artery; LM, left main stenosis; RCA, right coronary artery; CABG, coronary artery bypass graft.

**Figure 2:**
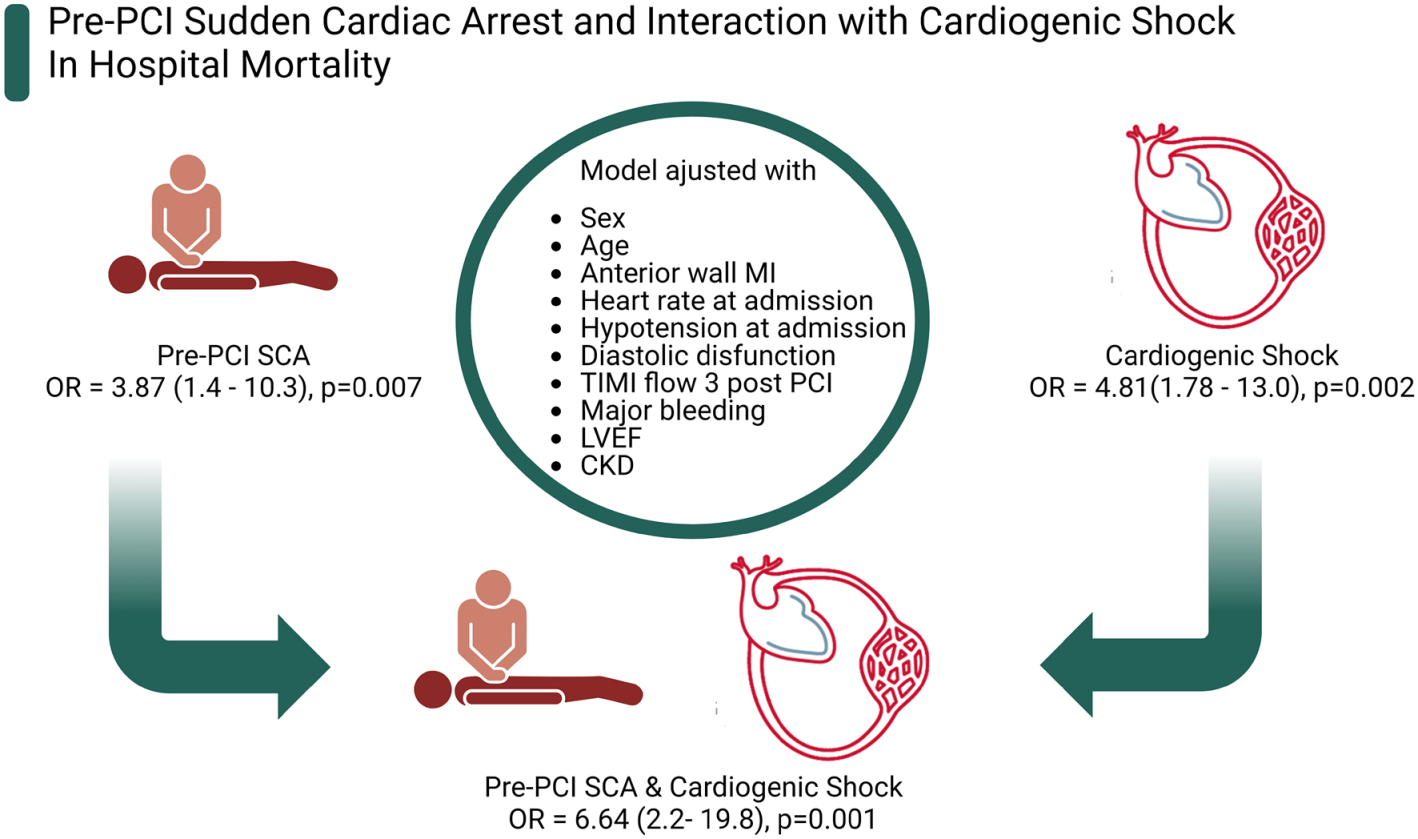
Pre-PCI sudden cardiac arrest and interaction with cardiogenic shock in hospital mortality. Figure created with BioRender.com.

The primary endpoint (in-hospital mortality) occurred in 36.8% of patients with pre-PCI SCA and 8.8% of those without SCA (*p* < 0.0001). In multivariate analysis anterior MI, cardiogenic shock, age, pre-PCI SCA, and lower ejection fraction remained significantly associated with in-hospital mortality (Table 2). Univariate analysis are described in Supplementary Table S2. When we analyzed the interaction between pre-PCI SCA and cardiogenic shock upon admission (Figure 2), each condition separately is associated with increased mortality risk (pre-PCI SCA: OR 3.87; 95% CI = 1.44–10.32; *p* = 0.007; CS: OR = 4.81; 95% CI = 1.78–13.00; *p* = 0.002), there is a further increase in mortality risk when both conditions are present (CS & pre-PCI SCA: OR = 6.64; 95% CI = 2.22–19.83; *p* = 0.001).

**Table 2:** Variables associated in-hospital mortality in multivariate analysis.

	OR	95% CI	Value of p
Male	1.34	0.74–2.43	0.33
Age (per year)	1.06	1.04–1.09	<0.0001
Anterior wall MI	1.76	0.92–3.36	0.08
Pre-PCI SCA	2.33	1.05–5.14	0.03
Cardiogenic shock	3.47	1.53–7.88	<0.01
CKD	2.03	0.78–5.27	0.14
Heart rate	1.01	0.99–1.02	0.14
Moderate/Severe diastolic disfunction	0.96	0.23–4.04	0.96
Moderate/Severe bleeding	3.56	0.96–13.14	0.056
TIMI 3 flow post PCI	0.73	0.37–1.44	0.37
LVEF, %	0.94	0.91–0.96	<0.0001
Hypotension	1.63	0.65–4.07	0.29

OR, odds ratio; CI, confidence interval; MI, myocardial infarction; PCI, percutaneous coronary intervention; SCA, sudden cardiac arrest; CKD, chronic kidney disease; LVEF, left ventricle ejection fraction.

When analyzed predictors of SCA in multivariate analysis, age (OR = 0.98; 95% CI = 0.98–0.99; *p* = 0.03) and cardiogenic shock (OR = 23.04; 95% CI = 13.98–37.95; *p* = <0.0001) remained significantly associated with this outcome (Table 3). Univariate analysis are described in Supplementary Table S3.

**Table 3:** Variables associated with pre-PCI sudden cardiac arrest in multivariate analysis.

	OR	95% CI	*p*-Value
Male	1.56	0.94–2.58	0.83
Age, per years	0.97	0.95–0.99	0.03
Anterior wall MI	1.28	0.81–2.00	0.28
Hypertension	0.88	0.53–1.46	0.62
Diabetes	0.63	0.63–1.46	0.10
Family history for CAD	0.94	0.94–1.74	0.86
Smoking	0.65	0.41–1.04	0.07
Previous ASA use	1.08	0.54–2.16	0.81
CKD	1.02	0.38–2.71	0.95
BMI, kg/m^2^	1.00	0.96–1.06	0.65
≥3 vessel disease	0.79	0.46–1.37	0.41
Previous MI	0.83	0.39–1.75	0.63
Cardiogenic shock	23.04	13.98–37.95	<0.0001

OR, odds ratio; CI, confidence interval; MI, myocardial infarction; CAD, coronary artery disease; ASA, acetylsalicylic acid; CKD, chronic kidney disease; BMI, body mass index.

**Figure 3:**
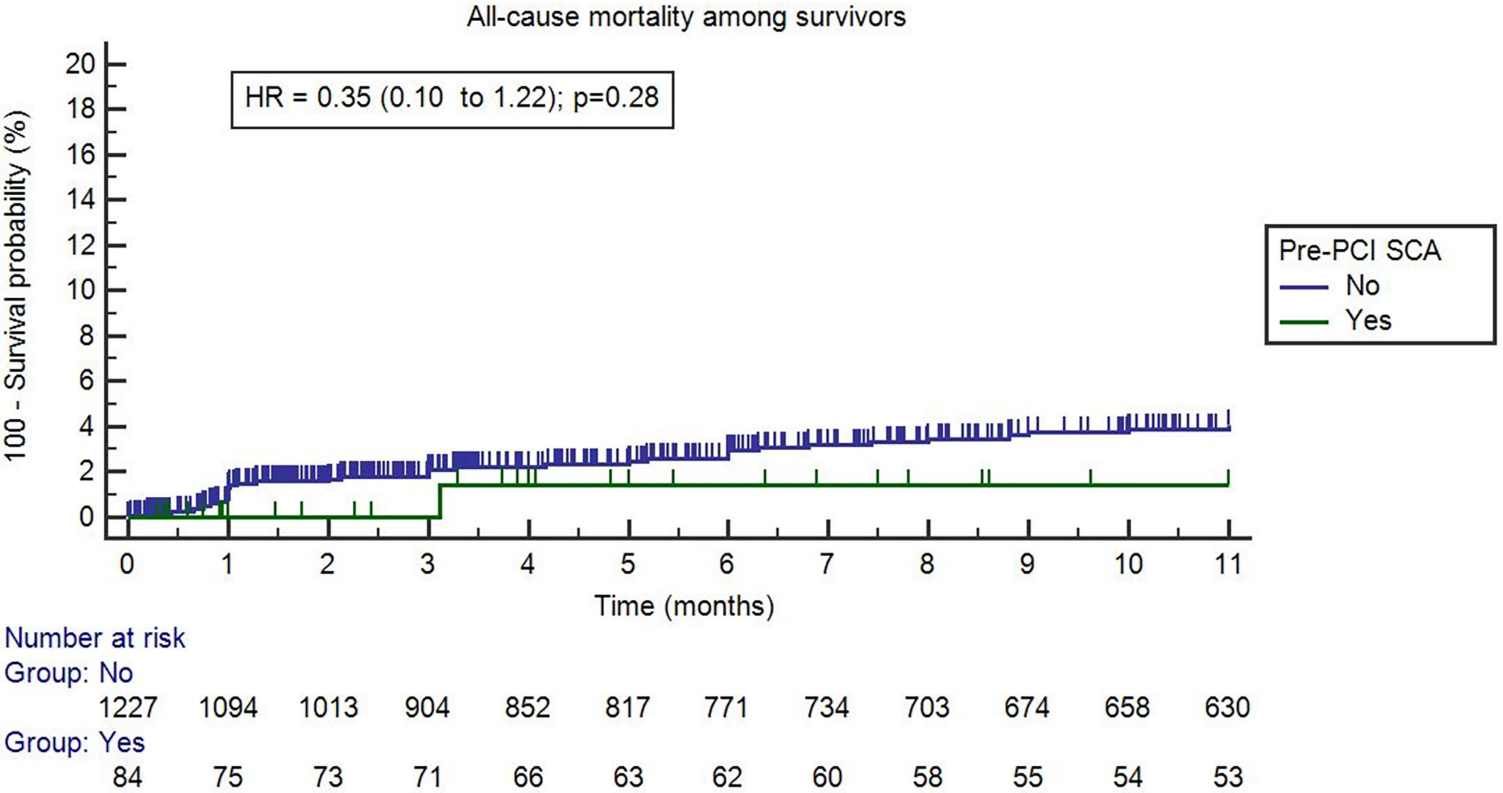
Time-to-event curves for all-cause mortality among survivors. Time “0” refers to hospital discharge. Event rates were calculated with the use of Kaplan–Meier methods.

After 12 months of follow-up, the overall mortality rates were similar between pre-PCI SCA survivors and the non-pre-PCI SCA group (1.2% vs. 3.1%, *p* = 0.32). During the follow-up period, cumulative event-free for all-cause mortality rates were comparable in pre-PCI SCA and non-SCA groups (Figure 3). The median overall time-to-event for all-cause mortality was 8.54 months. An analysis including all patients (survivals and non-survivals of in-hospital period) was included in Supplementary material – Supplementary Figure S1.

## Discussion

In this prospective cohort study evaluating STEMI patients submitted to primary PCI, the presence of pre-PCI SCA was strongly associated with higher in-hospital mortality, and the presence of acute heart failure and cardiogenic shock upon admission further increases mortality risk. Moreover, long-term mortality among pre-PCI SCA patients discharged alive was similar to non-SCA patients. To our knowledge, this is the largest study assessing SCA in STEMI patients in low and middle-income countries.

Clinical characteristics and initial management of STEMI patients are usually different between low-to middle-and high-income countries, as a consequence of the patient’s education, system organization, health funding, and other factors. Our cohort is no different, with a high incidence of pre-PCI SCA and increased in-hospital mortality in patients overall. Nevertheless, variables associated with pre-PCI SCA in our analysis are consistent with previous studies. In the APEX AMI trial, 5,745 patients were admitted with STEMI in 17 countries in North America, Europe and Oceania, and the occurrence of SCA was associated with a higher Killip classification (16). Karam et al. assessed 13,253 STEMI patients in the Paris area and found an independent association of anterior MI with a higher risk of SCA. Both anterior MI and higher Killip class are associated with a larger acute ischemic burden, increasing the risk of cardiac collapse and SCA (17).

In our cohort, the risk of pre-PCI SCA did not increase with the presence of classic risk factors of ischemic heart disease such as gender, family history, diabetes, hypertension and prior MI (18). Moreover, we found an inverse association between age and SCA. Despite some physiopathological mechanisms that could explain our findings such as a higher rate of ventricular fibrillation (VF) in patients <60 years in acute myocardial infarction or even the occurrence of atypical symptoms in younger patients, given the inherent nature of our observational study, this imbalance is most possibly due a survival bias, since older patients may be less likely to survive until hospital admission and more likely not forwarded to PCI as predicted outcome after SCA was deemed poor. (19–22) Conversely, Trepa et al. found no association with sex or age, but rather with the presence of diabetes, cerebrovascular diseases, and multivessel disease in particular (23). The combination of cardiogenic shock and sudden cardiac arrest was associated with higher in-hospital mortality than either entity alone in a cohort of STEMI patients, corroborating our findings. However, the presence of cardiogenic shock as a predictor of SCA must be interpreted with caution since cardiogenic shock can occur as a consequence of SCA (post-arrest shock) (24). Unfortunately, data on time of occurrence of cardiogenic shock (pre versus post SCA) were not available at our registry.

Survival rates in patients with pre-PCI SCA are increasing due to improvements in pre-hospital care, although in-hospital mortality remains high but quite variable across studies. Our study included an all-comers cohort of STEMI patients and found an in-hospital mortality of 36.8% in the SCA group, comparable to the average mortality of studies evaluating pre-PCI SCA. Dumas et al. analyzed 714 patients with SCA submitted to PCI and found hospital survival of 40% (3) Demirel et al. assessed the impact of pre-PCI SCA due to ventricular fibrillation or ventricular tachycardia in 4683 STEMI patients and found an in-hospital survival of 13.8%. Likewise, Orvin et al. and Bougouin et al. analyzed 300 and 116 patients, respectively, developing VF in the setting of acute coronary syndrome and found a higher risk of in-hospital mortality. However, like our findings, there was no correlation with long-term prognosis (21, 25, 26).

In our study, approximately 70% of the patients who presented pre-PCI SCA developed acute heart failure and cardiogenic shock. This is somehow expected, since the delayed return of spontaneous circulation predisposes failure of coronary reperfusion, sustained and refractory ventricular arrhythmias, hemodynamic instability, and a downward spiral of cardiogenic shock, further contributing to the increased mortality during hospitalization. Interestingly, the mortality risk was more than six times lower in the 30% of pre-PCI SCA patients who did not develop cardiogenic shock. Exploring this finding in larger cohorts may provide insights into patients’ characteristics associated with a better prognosis after SCA. Finally, when only analyzed pre-PCI SCA patients, cardiogenic shock upon admission was an independent predictor of in-hospital mortality. These findings are also comparable to previous studies evaluating STEMI-related SCA patients (17, 27).

Another important finding of our study is that, after hospital discharge, SCA survivors had a similar mortality rate after 12 months of follow-up compared to patients without SCA. This reassures that after the acute phase of SCA-related STEMI, the risk of adverse outcomes is similar when the reversible cause of SCA - STEMI - is resolved. This corroborates the findings of several studies evaluating out-of-hospital cardiac arrest and supports the lack of benefit of implanting cardioverter-defibrillator (ICD) devices in patients with a reversible cause (21, 25, 26, 28). Despite few studies, including a small randomized trial, suggesting a potential benefit of ICD implantation in high-risk patients (29, 30), current guidelines do not support this practice (31).

Some strengths and limitations are noteworthy. This study has limitations that are inherent to observational studies. Some data were obtained retrospectively, including previous atrial fibrillation, need for dialysis, and consciousness after SC, which can result in less reliable information. Data regarding point-of-care cardiac ultrasound at hospital admission was not available for analysis. Some echo parameters at an early assessment would be likely associated with the outcomes of the study. However, despite its single-center nature, this study represents a registry of consecutive and unselected patients presenting cardiac arrest coming from a tertiary referral hospital in the treatment of acute coronary syndromes, therefore the data shown are highly applicable to daily clinical practice.

In a cohort of consecutive patients admitted with STEMI, pre-PCI SCA was associated with higher in-hospital mortality, and its association with cardiogenic shock further increases mortality risk. Moreover, long-term mortality among pre-PCI SCA survivors was similar to non-SCA patients. Understanding characteristics associated with pre-PCI SCA may help to prevent and improve the management of STEMI patients.

## Data availability statement

The raw data supporting the conclusions of this article will be made available by the authors, without undue reservation.

## Ethics statement

The studies involving human participants were reviewed and approved by Research Ethics Committee of Hospital de Clínicas de Porto Alegre. The patients/participants provided their written informed consent to participate in this study.

## Author contributions

AT, RW, and SG: conceptualization. MN, JF, and AC: data curation. GM and GA: formal analysis. AT, GA, and GM: writing. MP and MW: supervision. All authors contributed to the article and approved the submitted version.

## Funding

This study was partially funded by Hospital de Clínicas de Porto Alegre (Fundo de Incentivo à Pesquisa e Eventos; FIPE/HCPA), Postgraduate Program in Health Sciences: Cardiology and Cardiovascular Sciences at the Federal University of Rio Grande do Sul (UFRGS), Conselho Nacional de Desenvolvimento Científico e Tecnológico (National Council for Scientific and Technological Development, CNPq) and Coordenação de Aperfeiçoamento de Pessoal de Nivel Superior (CAPES). The sponsors had no participation in the design, conduct of the study, preparation, analysis or approval of the manuscript. Guilherme Pinheiro Machado had a Doctoral degree fellowship from the Coordenação de Aperfeiçoamento de Pessoal de Nivel Superior (CAPES) – finance code 001. Marco Vugman Wainstein had a Research fellowship (Bolsa PQ) from the National Council for Scientific and Technological Development (CNPq).

## Conflict of interest

The authors declare that the research was conducted in the absence of any commercial or financial relationships that could be construed as a potential conflict of interest.

## Publisher’s note

All claims expressed in this article are solely those of the authors and do not necessarily represent those of their affiliated organizations, or those of the publisher, the editors and the reviewers. Any product that may be evaluated in this article, or claim that may be made by its manufacturer, is not guaranteed or endorsed by the publisher.
