# Supplementary material for: Pre-percutaneous coronary intervention sudden cardiac arrest in ST-elevation myocardial infarction: Incidence, predictors, and related outcomes
Source: Front Cardiovasc Med. 2023 Feb 16;10:1100187. doi: 10.3389/fcvm.2023.1100187 (PMC9978146; doi:10.3389/fcvm.2023.1100187)

SUPPLEMENTAL TABLES AND FIGURES

Pre-PCI sudden cardiac arrest in ST-elevation myocardial infarction: incidence, predictors and related outcomes

Machado, GP; Theobald, AL; Araujo, GN; Donelli, A; Wainstein, RV; Fracasso, JF; Niches, M; Chies A; Goncalves, SC; Pimentel, M; Wainstein, MW.

Page 2: Supplemental Table S1

Page 3: Supplemental Table S2

Page 4: Supplemental Table S3

Page 5: Supplemental Figure S1

Supplemental Table S1: Number of patients for which the respective data was available.

Clinical Characteristics	Valid (n=1493 | *133)
**Age, y**	1493
**Male**	1493
**BMI**	1354
**Hypertension**	1492
**Diabetes**	1492
**Previous ASA use**	1488
**Previous AMI**	1492
**Previous PCI**	1487
**Previous Stroke**	1492
**Previous HF**	1492
**Previous COPD**	1492
**Previous CKD**	1492
**Peripheral Vascular Disease**	1492
**Family history**	1478
**Smoking, current or previous**	1493
**Atrial Fibrillation**	1493
**Temporary Pacing**	1484
**Cardiogenic Shock**	1493
**Admission status**	1492
**SCA location**	132*
**Assisted SCA**	133*
**Rhythm**	120*
**ECG after SCA**	104*
**ROSC, *min***	122*
**Conscious after resuscitation**	123*
**Heart rate, bpm**	1452
**SBP, mmHg**	1461
**SBP < 90 mmHg**	1470
**Pain-to-door, *min***	1416
**Door-to-balloon, min**	1292
**LVEF, %**	1339
**Diastolic Disfunction**	1165
**RV disfunction**	1339
**Gusto Bleeding Classification**	1493
**Mechanical complication**	1493
**Dialysis in first 24h of admission**	1493
**Thrombolysis**	1475
**CABG**	1493
**Arrythmias after 48h**	1493
**Radial Access**	1486
**TIMI pre-PCI**	1373
**TIMI post-PCI**	1437
**Anterior wall MI**	1490
**Infarct related artery**	1481
**Number of vessels with severe stenosis**	1492
**No-reflow**	1493
**Successful rates**	1460

Table S2: Univariate analysis for in-hospital mortality modeling.

	OR	95% CI	p-value
**Male**	0.77	0.56 – 1.08	0.13
**Age,** *(per year)*	1.05	1.03 - 1.06	<0.0001
**Anterior Wall MI**	2.00	1.44 - 2.79	<0.0001
**Pre-PCI SCA**	6.08	4.08 - 9.07	<0.0001
**Cardiogenic Shock**	11.26	7.69 - 16.50	<0.0001
**CKD**	3.11	1.79 - 5.41	<0.0001
**Heart Rate**	1.02	1.01- 1.03	<0.0001
**Moderate/Severe Diastolic Disfunction**	2.01	0.68 - 5.89	0.20
**Moderate/Severe Bleeding**	9.81	4.99 - 19.28	<0.0001
**TIMI 3 flow post PCI**	0.26	0.17 - 0.39	<0.0001
**LVEF,** *%*	0.93	0.91 - 0.95	<0.0001
**Hypotension**	7.04	4.61 - 10.74	<0.0001

Table S3: Univariate analysis for pre-PCI sudden cardiac arrest modeling.

	OR	95% CI	p-value
**Male**	1.23	0.76 – 1.63	0.54
**Age,** *per years*	0.98	0.97 – 1.00	0.10
**Anterior Wall MI**	1.60	1.11 – 2.98	0.01
**Hypertension**	0.81	0.56 - 1.16	0.26
**Diabetes**	0.98	0.66 - 1.47	0.94
**Family History for CAD**	0.88	0.53 - 1.45	0.63
**Smoking**	0.67	0.46 - 0.96	0.02
**Previous ASA use**	1.01	0.63 – 1.62	0.96
**CKD**	1.31	0.61– 2.81	0.47
**BMI,** *kg/m²*	1.02	0.98 - 1.06	0.29
**≥3 vessel disease**	0.75	0.48 – 1.17	0.21
**Previous MI**	1.03	0.61 – 1.74	0.88
**Cardiogenic Shock**	18.01	11.96-27.12	<0.0001

Supplemental Figure S1 - Time-to-event curves for all-cause mortality. Time “0” refers to hospital admission. Event rates were calculated with the use of Kaplan–Meier methods.

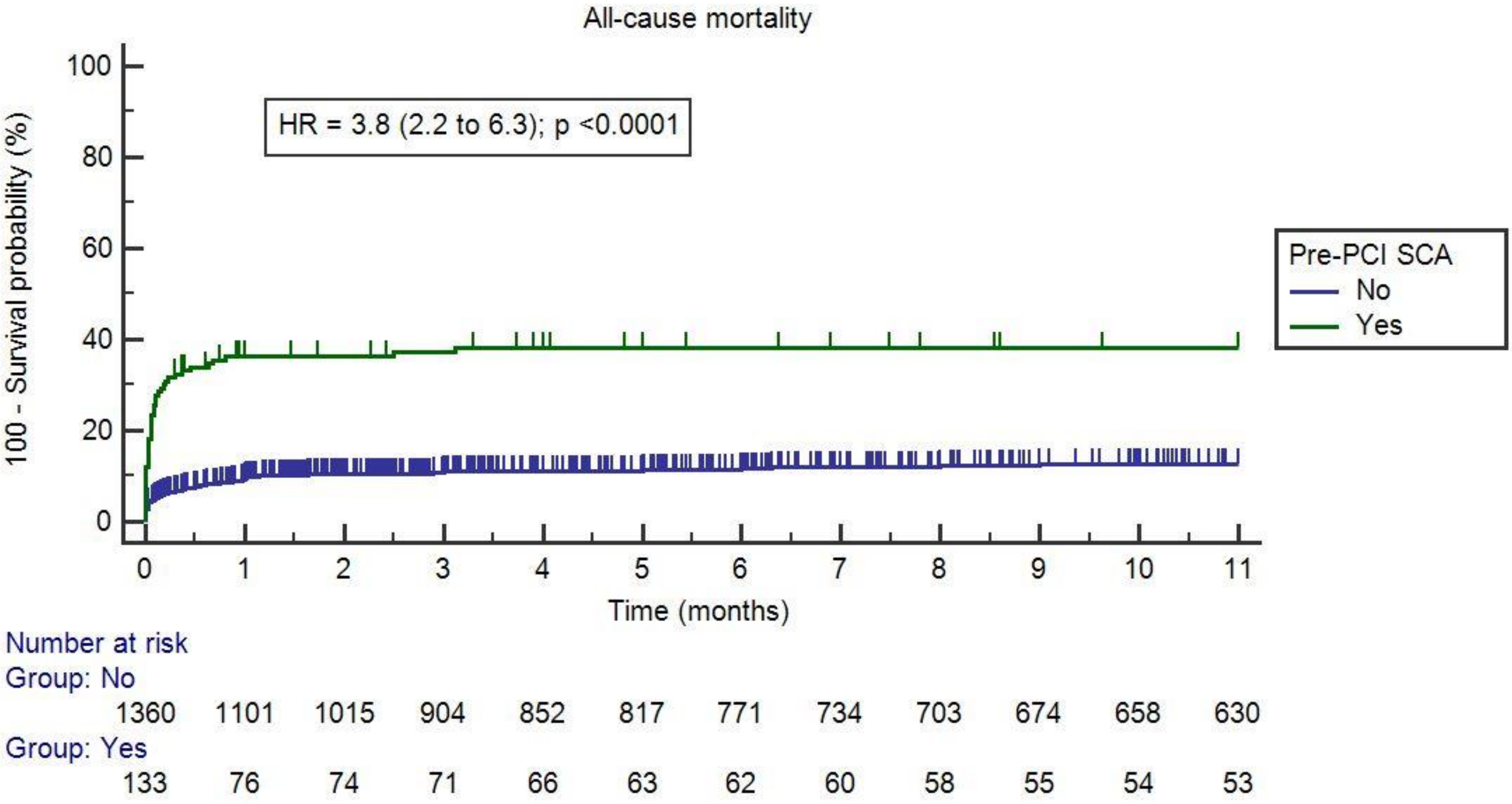

Supplement: Supplementary file 1 [file Data_Sheet_1.PDF]
